# Comparison of Point Cloud Registration Techniques on Scanned Physical Objects

**DOI:** 10.3390/s24072142

**Published:** 2024-03-27

**Authors:** Menthy Denayer, Joris De Winter, Evandro Bernardes, Bram Vanderborght, Tom Verstraten

**Affiliations:** 1 Robotics & Multibody Mechanics Group, Vrije Universiteit Brussel, Pleinlaan 9, 1050 Brussels, Belgium; joris.de.winter@vub.be (J.D.W.); evandro.bernardes@vub.be (E.B.); bram.vanderborght@vub.be (B.V.); 2 Flanders Make, Pleinlaan 9, 1050 Brussels, Belgium; 3 IMEC, Pleinlaan 9, 1050 Brussels, Belgium

**Keywords:** point cloud registration, digital twins, CAD model alignment, point cloud datasets

## Abstract

This paper presents a comparative analysis of six prominent registration techniques for solving CAD model alignment problems. Unlike the typical approach of assessing registration algorithms with synthetic datasets, our study utilizes point clouds generated from the Cranfield benchmark. Point clouds are sampled from existing CAD models and 3D scans of physical objects, introducing real-world complexities such as noise and outliers. The acquired point cloud scans, including ground-truth transformations, are made publicly available. This dataset includes several cleaned-up scans of nine 3D-printed objects. Our main contribution lies in assessing the performance of three classical (GO-ICP, RANSAC, FGR) and three learning-based (PointNetLK, RPMNet, ROPNet) methods on real-world scans, using a wide range of metrics. These include recall, accuracy and computation time. Our comparison shows a high accuracy for GO-ICP, as well as PointNetLK, RANSAC and RPMNet combined with ICP refinement. However, apart from GO-ICP, all methods show a significant number of failure cases when applied to scans containing more noise or requiring larger transformations. FGR and RANSAC are among the quickest methods, while GO-ICP takes several seconds to solve. Finally, while learning-based methods demonstrate good performance and low computation times, they have difficulties in training and generalizing. Our results can aid novice researchers in the field in selecting a suitable registration method for their application, based on quantitative metrics. Furthermore, our code can be used by others to evaluate novel methods.

## 1. Introduction

Point cloud registration (PCR) is used in applications like building information modelling [1], augmented reality authoring [2] and robotics [3]. The problem of PCR consists in finding the (rigid) transformation between two point clouds, the source and the template, minimizing a cost function. These point clouds can be sampled from available CAD models or generated using stereovision [4] and laser-scanning techniques, including LiDAR [5]. In practical applications such as manufacturing, a combination of CAD models and (partial) scanning data is often used.

A closed-form solution exists when correspondences between the two point clouds are exactly known [6]. This is the case, for example, when using synthetic data, generated on a computer. However, these correspondences are unknown and not exact when working with real-world scans or point clouds captured by different sensors. Synthetic datasets approximate this by adding Gaussian noise [7] or removing a part of the object (partiality) [8,9]. Still, these approximations fail to capture real-world 3D-scans, as they lack details like the rounding of sharp corners, deformations, density variations or slight scaling. We believe the community could benefit from a comparison with point clouds sampled from CAD models and generated using laser-scanning. However, such comparisons are lacking in the existing literature.

Review papers on PCR techniques, metrics and datasets are presented in the literature [10,11,12,13,14,15]. These typically use synthetic datasets [12], such as the Stanford Bunny [16]. Comparisons on real-world scans exist for LiDAR data [5], but existing datasets are mostly limited to indoor and outdoor environments [17,18], instead of specific objects [19,20,21]. Aside from the MVTEC ITODD dataset [22], which focuses on industrial object scans, other datasets mainly include everyday objects or environments. To address this gap, we collect new real-world data, based on the Cranfield benchmark [23], containing basic geometries. These basic objects facilitate the creation of clear 3D scans while introducing challenges such as symmetric solutions, noise and partiality.

Aligning the captured point clouds with their CAD models is often done using the standard ICP method in the literature [4,24,25,26]. However, this typically involves using a (fully) scanned template model [24,26,27], instead of sampling from a CAD model. While popular, ICP’s performance is sensitive to the initial pose of both objects and can easily fall into local minima solutions [10]. Thus, there is a need to look for other, more robust registration methods.

Classifications of PCR methods have been proposed in the literature [28]. Ref. [29] compares different feature descriptors for ICP-based methods and RANSAC-based methods (SAC-IA). They find the Fast Point Feature Histogram (FPFH) to be accurate and fast. For this reason, it is also used in this paper. However, we extend the comparison to include other methods, like FGR and deep learning methods. Comparing several techniques is crucial to expand beyond the basic ICP method in practical applications. As indicated by [28,30], there is still a large reliance on classical methods like ICP and NDT, while benchmarks for learning-based methods and pretrained models for real-life scenarios are lacking. The comparison by [31] focuses on RANSAC-based methods and inlier IG-methods. Ref. [32] includes recent deep learning methods such as SpinNet [33] and a graph-based method, TEASER [6]. However, the comparison is again performed on range scans, instead of solving the CAD alignment problem. Finally, ref. [34] compares deep-learning-based registration methods based on previously published metrics. We compare six different registration methods, including RANSAC, FGR, PointNetLK and RPMNet, which are important, well-known global and learning-based registration techniques [35]. Alternative methods exist in the literature, like probabilistic methods (Deep-GMR [36], NDT [37], CPD [38]), graph-based methods (TEASER [6]) and other learning-based methods (DeepPro [39], SpinNet [33], REGTR [40]). These are considered to be out of scope for this paper. We focus instead on classical and learning-based techniques. However, the created code allows others to evaluate their performance using the same methodology on a given dataset.

In this paper, CAD model alignment is used to compare six popular registration methods, including GO-ICP [8], RANSAC [41], FGR [42], PointNetLK [43], RPMNet [9] and ROPNet [44]. The point clouds are generated from an available CAD model and a 3D-scan, created using the Intel RealSense D435i camera. New scans are created based on the Cranfield benchmark dataset. The scans contain noise and outliers, which are typical challenges in PCR, to verify performance in real-world applications. The following assumptions are made:

1. Each point cloud consists of a single object that is already segmented from the environment. However, we adapt the quality of the cutout as a parameter.
2. We already assign each point cloud a label corresponding to the represented object.
3. We do not consider large deformations and shearing [45,46].

The main contributions of this paper are:

1. A comparison of the performance of six registration methods, applied on real-world scans of 3D-printed objects, with relatively basic geometries, and their CAD models.
2. A dataset consisting of a series of real-world scans, based on the Cranfield benchmark dataset [23] with available ground-truth estimation. The Python code, used to run the experiments, and the point cloud scans with their ground truth, are available at https://github.com/Menthy-Denayer/PCR_CAD_Model_Alignment_Comparison.git (accessed on 14 February 2024).

The remainder of this paper is organized as follows. Section 2, Methodology, details the methodology, including the chosen registration methods, metrics, datasets and ground-truth estimation used to assess the performance. The results of the experiments are presented in Section 3, Results, and discussed in Section 4, Discussion. Finally, Section 5, Conclusion, contains conclusions and future work opportunities.

## 2. Methodology

### 2.1. Registration Methods

We selected registration methods based on the following criteria:

- Robustness to noise. Three-dimensional cameras were used to create point clouds. Working in nonoptimal lighting conditions or cluttered environments results in measurement errors and noise. This leads to deformations of the scan compared to the real object, making it more difficult to find correspondences for the registration methods.
- Robustness to partiality. Since we used a single camera in this paper, the object was only visible from one perspective. As a result, the captured point cloud was incomplete, missing the parts of the object, which the camera could not see.
- Limited computation time. For real-time applications, the computation time for the registration process has to be limited. Timing can also be an important aspect to consider when training the learning-based methods.
- Ability to generalize to different objects. The different methods have to work on a wide variety of objects to be widely applicable. Learning-based methods are trained on available CAD models and risk overfitting. Non-learning-based methods can generalize better, which may come at the cost of a lower performance.

These criteria are typically used in the literature to describe the advantages and disadvantages of the different algorithms. We favoured open-source codes to adapt the methods into the comparison framework. Open3D [47] provides an open-source library including the RANSAC, FGR and ICP registration methods. These are standard and popular methods, often used in real-world applications [3,26,48]. Furthermore, we selected learning-based methods for their improved robustness and accuracy when dealing with extensive real-world scans [10,39,49]. Additionally, there is a need to benchmark these methods in the literature [30]. The selected techniques are shortly discussed in Section 2.1.1 and Section 2.1.2.

#### 2.1.1. Non-Learning-Based Methods

Non-learning-based methods do not have to be trained and are therefore quick to set up and use. We implemented GO-ICP from the author’s code [8], and we used the Open3D implementation for RANSAC and FGR [47].

- GO-ICP [8] improves upon the standard ICP method by finding the global optimum solution. ICP-based methods solve the registration problem by minimizing a cost function. These algorithms typically establish correspondences based on distance. GO-ICP is robust to noise. However, the method tends to be slow.
- RANSAC [41] is a global registration method, often used in scene reconstruction [48]. It uses the RANSAC algorithm to find the best fit between the template and source point clouds. Features are extracted using Fast Point Feature Histogram (FPFH) [50], which is a point-based method [32]. RANSAC is robust to outliers and noise and does not require any training process. However, it requires a preliminary step for feature extraction and there are multiple parameters to tune.
- FGR [42] is a fast registration method, requiring no training. It also uses FPFH to extract features but does not recompute the correspondences during the execution. It can perform partial registration but is more sensitive to noise.

We selected GO-ICP as it provides robust, high-accuracy results. Thus, it is interesting to compare its outcomes to methods like FGR and RANSAC, which are much faster, but less reliable.

#### 2.1.2. Learning-Based Methods

Learning-based methods are trained using a dataset to extract features and compute the transformation matrix. The training process consists of creating many iterations of a template and a transformed source. Each time the registration problem is solved and compared to the ground-truth solution. An error is then computed to adjust the weights of the network. We implemented PointNetLK and RPMNet from available codes [51], while we took ROPNet from the author’s code [44].

- PointNetLK [43] is a learning-based method. It uses PointNet to generate descriptors for each point. This information is then used to compute the transformation matrix through training. The method is robust to noise and partial data. However, the performance drops when the method is applied to unseen data and for large transformations.
- RPMNet [9] is another learning-based method. It combines the RPM method with deep learning. RPM itself builds upon ICP, using a permutation matrix to assign correspondences. The transformation matrix is computed using singular value decomposition (SVD). RPMNet is robust to initialization and noise, and also works for larger transformations. RPMNet is, however, reported by [9] to be slower than other methods like ICP or DCP.
- ROPNet [44] is a learning-based method, created to solve the partial registration problem. First, a set of overlapping points is established. Afterwards, wrong correspondences are removed, turning the partial-to-partial into a partial-to-complete problem. Finally, SVD is used to compute the transformation matrix. It is robust to noise and can generalize well.

PointNetLK forms an important milestone for deep-learning-based PCR methods, while RPMNet and ROPNet are promising novel approaches. We selected these methods as each one approaches the problem of PCR differently, resulting in different training capabilities and accuracies.

### 2.2. Metrics

We used four groups of metrics to compare the registration methods. The first group of metrics expressed the errors in degrees and a unit of length. They were the easiest to interpret and yielded a direct evaluation of the rigid transformation. These metrics included the mean absolute error (MAE) [9], mean relative error (MRE) [13] and root-mean-square error (RMSE) [36]. All metrics could express both translational and rotational errors, in a unit of length and degrees, respectively.

A second group of metrics evaluated the accuracy of the alignment. This included the recall [36] metric and the coefficient of determination $R^{2}$ [52]. The metrics were expressed as a number between zero and one or as a percentage. The better the alignment, the higher their value.

**Figure 1:**
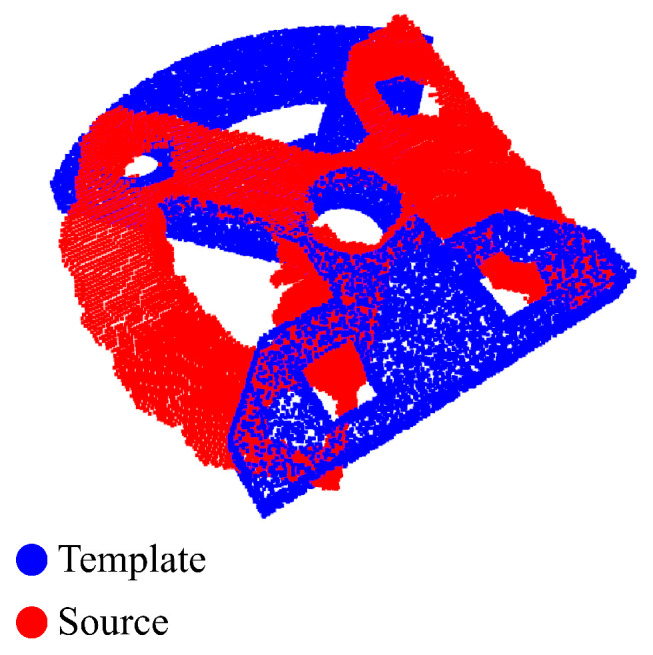
An example of a solution with a negative $R^{2}$ value. The estimated transformation (red) does not overlap the template (blue). The result shown is for the base-top plate object, after applying PointNetLK.

In some cases, the registration may lead to unsatisfactory results. In these cases, the absolute values of the errors are not as relevant. However, it is interesting to save the number of failure cases [6,21], which made up the third group of metrics, and the scans for which they occurred. Thus, whenever a result met the condition of $R^{2}<0$ or $MRAE>120^{\circ }$, it was considered to be a failure. An example of a negative $R^{2}$ result is shown in Figure 1. The condition aims at removing only the extreme cases of misalignment, where the found transformation is small. Thus, in these cases, ICP refinement cannot correct the results.

Finally, we also recorded the registration time as a metric. It was counted whenever the method is called upon, with the source and the template already loaded, until the transformation matrix was returned.

We implemented the evaluation of the results in Python. The code can be found at https://github.com/Menthy-Denayer/PCR_CAD_Model_Alignment_Comparison.git (accessed on 14 February 2024).

### 2.3. Materials

We used an Intel (USA) RealSense D435i camera to capture the point cloud scans. It can capture depth at 30 cm, yielding high-accuracy scans for the considered objects. The datasheet indicates an absolute error (z-accuracy) of ±2% for objects captured within 2 m from the camera. The spatial noise (RMSE) is less than 2%. The D435i camera has a 1280 × 720 depth resolution. We processed the captured point clouds manually using the RealSense viewer application, separated them from the environment and labelled them. We sampled the template point clouds from the corresponding CAD files with twice the number of points from the source (captured point cloud). We did this to obtain a similar point cloud density, considering partiality. The template was scaled to match the size of the captured point cloud.

**Figure 2:**
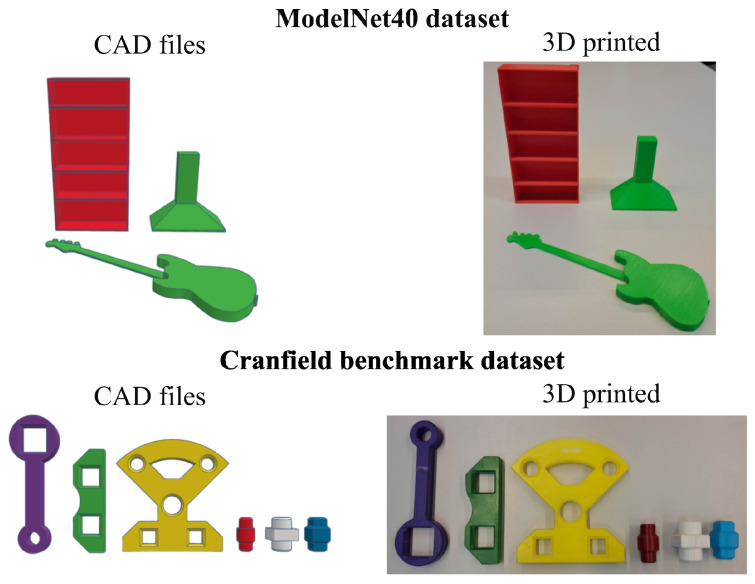
Objects from the ModelNet40 dataset and Cranfield benchmark used during the experiments.

**Figure 3:**
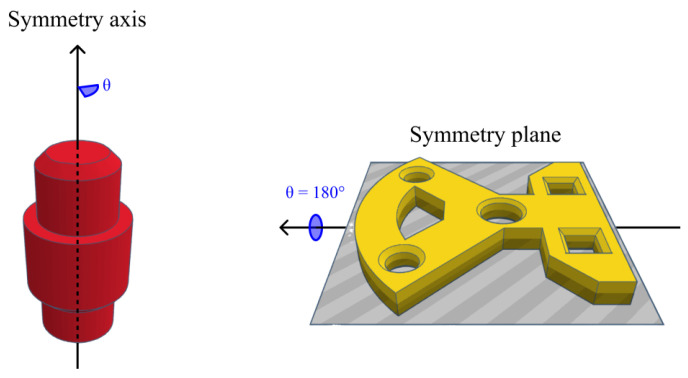
Objects from the Cranfield benchmark dataset contain a symmetry axis or symmetry plane, resulting in an infinite number of ground-truth solutions.

For the experiments, we used two datasets (Figure 2): the Cranfield benchmark [23] and the ModelNet40 [53] dataset. The Cranfield benchmark is used to assess, for example, robotic peg-in-hole (PIH) manufacturing operations. It contains six unique objects with basic geometries. The objects all have at least one symmetry axis, as shown in Figure 3. This means multiple ground-truth solutions exist, which were considered in the comparison. We 3D-printed the objects to reduce reflections, as these were not considered in this paper. The largest and smallest objects had a characteristic length of 22 cm and 6 cm, respectively.

The ModelNet40 dataset is typically used to evaluate point cloud registration methods on synthetic data. The dataset consists of multiple models in 40 categories of objects. We selected and 3D-printed three objects with simple geometries. Training data were also available for the learning-based methods [51].

**Figure 4:**
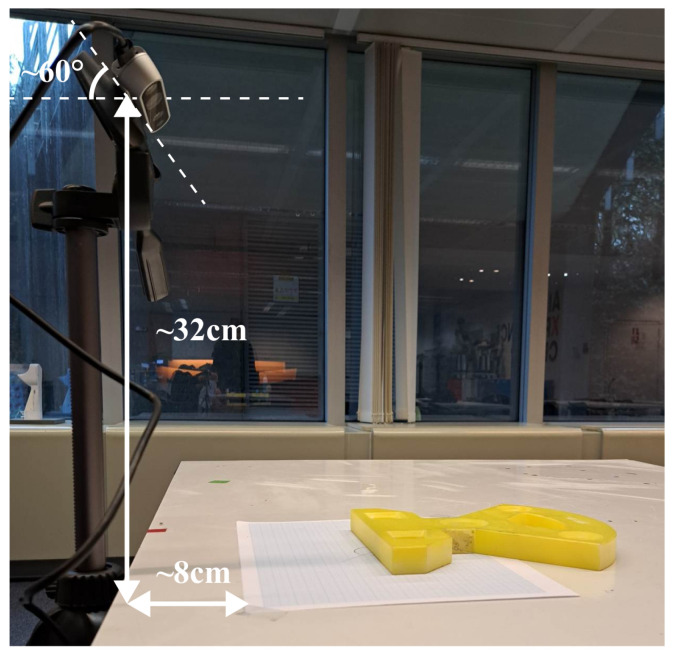
The experimental setup used for capturing the point clouds. The 3D camera is fixed. The paper is used as a reference for orienting the 3D-printed objects.

We placed the objects flat on a light table, as shown in Figure 4, in different orientations. The camera was fixed above the table. Depending on the object, we brought the camera closer or farther to obtain a clear point cloud scan. The objects were angled at $45^{\circ }$ or $90^{\circ }$. We only added scans when the general shape of the object was sufficiently recognizable.

We also used the datasets for training the learning-based methods, selected in Section 2.1.2. The training data were generated synthetically, as creating a sufficiently large dataset with real scans is very time-intensive. We sampled point clouds from the CAD models and randomly transformed them according to literature guidelines [7,9,43,44,52,54]. Additional variations were introduced in the data by adding noise, partiality, a floor or a combination. Due to convergence issues, not all methods could be trained on all datasets. All methods were trained on the normal and noisy (σ=0.01) datasets. RPMNet and ROPNet were trained on partial data, where 50% and 70% of the points were retained, respectively. The same methods were trained on the floor, noisy and partial dataset, with a noise level of 0.01 and 70% of points retained.

### 2.4. Ground-Truth Estimation

**Figure 5:**
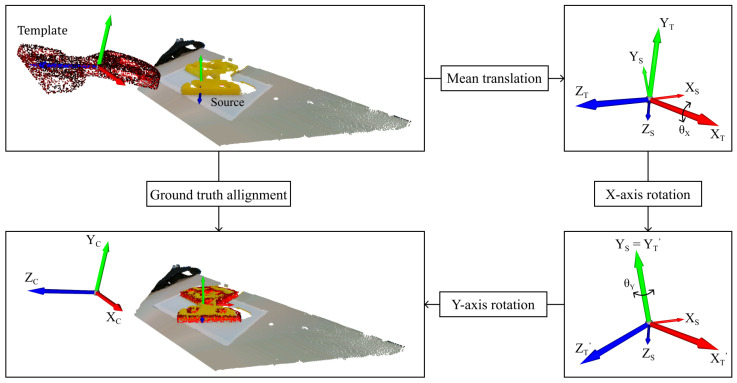
Steps performed to estimate the ground-truth estimation. The red and yellow point clouds represent the template and source, respectively. $X_{T}Y_{T}Z_{T}$ is the template’s and $X_{S}Y_{S}Z_{S}$ the source’s coordinate system.

To compute the metrics from Section 2.2, we needed to estimate the ground truth. The process was based on using known information, like the object’s orientation on the table, estimated values such as the normal vector on the table and finally, a visual correction for the translation [6]. We placed the 3D camera parallel to the table, which meant the x-axis of the template was too. Figure 5 shows the steps, which are detailed below.

1. We centred both point clouds on the origin by subtracting their mean.
2. Using an estimation of the normal vector on the table, we performed a rotation around the template x-axis, aligning the y-axes of both objects.
3. Given the known rotation of the object on the table, we performed a final rotation around the new y-axis.
4. We performed visual corrections for the rotation and mainly translation, similar to [6].

Appendix A (Table A1) details the validation of the ground truth, showing an accuracy of around 2 mm and $3^{\circ }$. The processed point clouds and their ground-truth transformations are available at https://github.com/Menthy-Denayer/PCR_CAD_Model_Alignment_Comparison.git (accessed on 14 February 2024).

### 2.5. Registration Parameters

Each registration method has a range of parameters that can be tweaked. The considered parameters are given in Table 1 for each method.

The zero-mean parameter refers to the centring of the point clouds, which reduces the transformation size. This parameter was considered as it is a simple pre-processing step, removing the offset from the camera.

For GO-ICP, the MSE threshold and trim fraction need to be defined. The MSE threshold determines the convergence criteria. The trim fraction determines the fraction of outliers to be removed.

The voxel size is a simple filtering technique, used to downsample the point clouds. It takes the average of all points inside a small voxel, with the voxel size indicating its scale. As this parameter reduces the number of points in the point cloud, it is interesting to consider its effect on the computation time.

**Figure 6:**
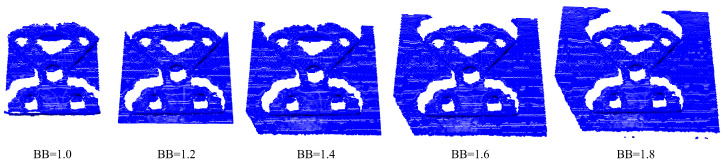
Effect of adapting the bounding box to increase the information around the object in the point cloud.

We adapted the bounding box to simulate the effect of different cutouts around the object, as in Figure 6. The larger the bounding box, the more environmental information is included in the point cloud. However, methods that can work for larger bounding boxes require less pre-processing. This is especially interesting in real-time applications. Furthermore, the maximal bounding box places additional requirements on preliminary object detection and filtering steps.

Finally, training models were varied for the learning-based methods. We used the Cranfield benchmark first to train the methods, as mentioned in Section 2.3. Pre-trained ModelNet40 training models were compared to the results achieved using the Cranfield benchmark.

Finally, we used ICP refinement to refine the results, similar to [6]. ICP [43] is often used in applications for its low computation time and simplicity. The main disadvantages of ICP include its lower performance for larger transformations and susceptibility to local minima.

### 2.6. Data Processing

**Figure 7:**
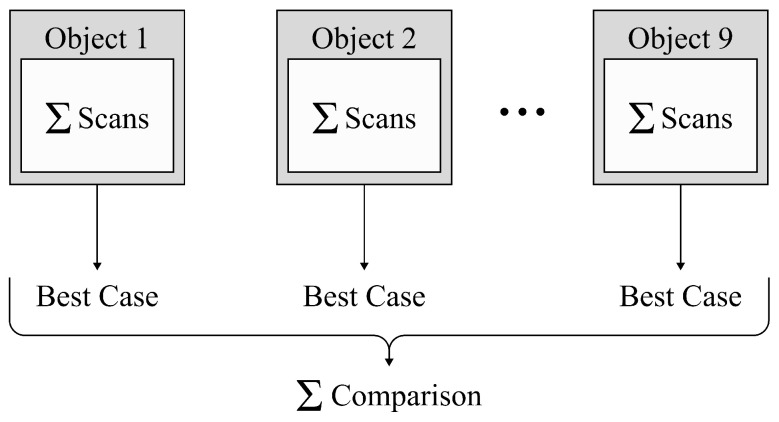
For each object, the results of the different scans were averaged (Σ). The best cases for each object, in terms of the parameters, were selected and averaged, to compare the different methods.

We applied the registration methods, selected in Section 2.1, on the created point cloud scans, to align them with their templates. For each object, we created multiple scans. The alignment was repeated several times to verify whether the same results were obtained. Furthermore, we varied several parameters for each registration method, as mentioned in Section 2.5. From these experiments, we selected the parameters leading to the best result, over the performed experiments, per object. This means the lowest values for the MAE, MRE, RMSE and number of failure cases and the highest values for the recall and $R^{2}$ metric. We then averaged the metrics for a final comparison of the methods. Figure 7 gives a schematic overview.

The standard deviation σ is the square root of the sum of experimental variances, weighted by the number of samples, not considering the failure cases. For the number of failure cases and time, we used the standard formula for the variance, with the sum taken over the different objects instead.

We ran our experiments on an HP Omen (USA) Windows laptop with an NVIDIA GeForce RTX 3070 GPU and AMD Ryzen 7 processor.

## 3. Results

### 3.1. Training Validation Results

We validated the learning process on a test dataset for the Cranfield models. Table 2 gives an overview. Since all points of the point clouds were rescaled to fit into a unit sphere, to comply with training standards [7,9,43,44,52,54], the synthetic objects were on the order of metres, compared to centimetres for the real, 3D-printed objects. Table 2 shows a largest error of 25.76 mm, which corresponds to 1.2% of the largest object’s characteristic length. Training validation indicated a good training convergence for all datasets. However, the recall values were lower, specifically for RPMNet and ROPNet, compared to PointNetLK. $R^{2}$ values were 1.00, indicating a perfect overlap. However, when working with large numbers, this metric is more prone to numerical rounding errors.

### 3.2. PCR Methods Comparison

We compared the methods by selecting a representative metric for key criteria, such as precision, variance, speed, generalizability and required pre-processing, as shown in Table 3. An overview of all metrics for each method can be found in Appendix B (Table A2).

The zero-mean method had little effect on the results. The best case was used for each method. GO-ICP, RPMNet and ROPNet were not centred (nonzero mean), while RANSAC and FGR were centred (zero mean). PointNetLK centred the point clouds automatically.

The lower the MSE threshold, the more accurate the results. As the value increased, the number of failure cases rapidly rose until all scans led to unsatisfactory results. However, the registration time increased on average by 10 s when lowering the threshold from $10^{-1}$ m to $10^{-5}$ m, with the largest object taking 67 s to solve. The trim fraction had little effect on the results, as the point clouds were already cleaned in pre-processing.

**Figure 8:**
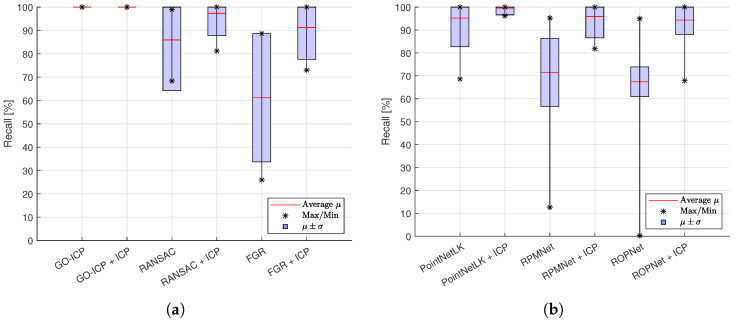
Average recall metric, with average maximal and minimal values indicated over the different objects, as well as the region of variation μ±σ. (**a**) Non-learning-based methods. (**b**) Learning-based methods.

**Figure 9:**
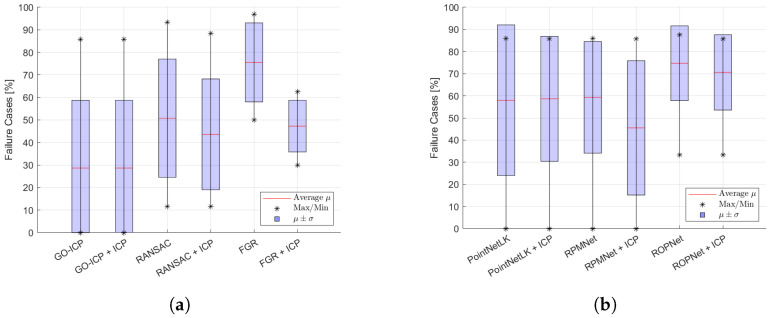
Average number of failure cases, with average maximal and minimal values indicated over the different objects, with the region of variation μ±σ. (**a**) Non-learning-based methods. (**b**) Learning-based methods.

The $R^{2}$ value and recall indicate the precision, as shown in Table 3 and Figure 8, respectively. GO-ICP achieved the highest accuracies, where a successful alignment usually coincided with recall values of 100% and $R^{2}=0.98$. RANSAC, PointNetLK and RPMNet, combined with ICP refinement, also resulted in precise alignments, though with increased variance, as seen in Figure 8. FGR was less precise, but one of the faster methods, as shown in Table 3. ROPNet scored well in terms of the recall and $R^{2}$ metrics, but led to a high number of failure cases, as shown in Figure 9, even after applying refinement. Figure 8 shows the effect of applying ICP refinement. For all methods, the recall metric improved significantly, while variances dropped. GO-ICP was the only exception, where ICP did not significantly improve the results.

**Figure 10:**
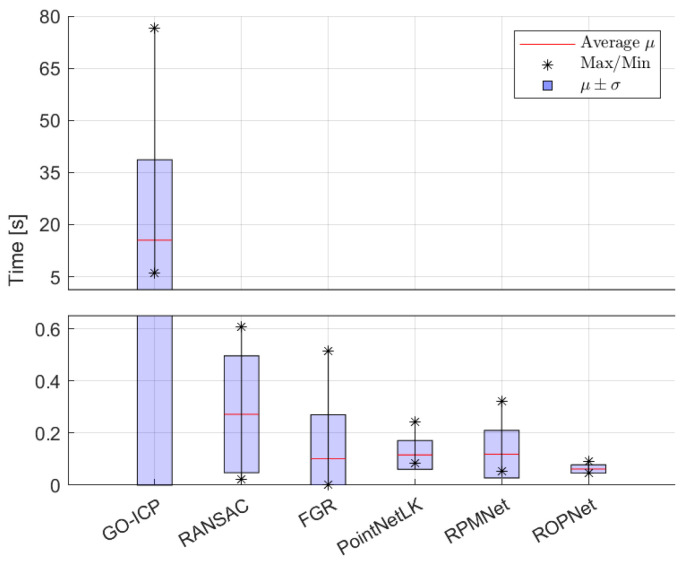
Average computation time for all methods, with average maximal and minimal values indicated over the different objects, with the region of variation μ±σ. GO-ICP leads to much higher registration times, hence the jump in values.

Computation times were highest for GO-ICP, as shown in Figure 10. The other registration methods took on average less than 1 s to solve. FGR was among the fastest methods on average but showed a larger spread. The learning-based methods were quick to solve the registration problem.

Figure 9 presents the number of failure cases. A large variation is visible for all methods over the different objects and scans. On average, GO-ICP led to the lowest number of failure cases, as also indicated in Table 3. In contrast, ROPNet showed the highest number of failure cases on average. Refinement had a small effect on that metric.

Finally, Table 3 highlights the pre-processing criterion. This refers to the required cleaning of the initial point cloud for the method to work successfully. The recall metric for a bounding box 80% larger than the cleaned-up point cloud was used as a representative metric. GO-ICP failed to converge when too much background clutter was included. PointNetLK and FGR yielded a result, though the registration quality was strongly reduced as the bounding box was increased, with recall values of only 0% and 12.65%, respectively. RANSAC and RPMNet both led to recall values above 20%. However, the methods performed the best when the point cloud was filtered from outliers and environmental clutter.

## 4. Discussion

### 4.1. Registration Parameters

The voxel size parameter significantly affected the registration results for RANSAC, FGR, ROPNet and RPMNet. For these methods, the recall metric dropped from >80% to <20%, for a change in the voxel size of only 2 mm. The parameter also did not show a clear trend. The optimal voxel size changes depended on the object, method and metric. PointNetLK was more indifferent to the voxel size, as the results remained consistent for almost all objects. The best results were found for a voxel size of around 1 cm. Finally, we combined GO-ICP with the voxel size. However, it is important to adapt the MSE threshold parameter accordingly, otherwise, the method no longer converges. Adapting the voxel size can improve the results for a higher MSE threshold. Most objects, leading originally to a zero recall, resulted in a recall of >60% after adapting the voxel size.

We found that the registration accuracy typically decreased with an increasing bounding box. Some methods, such as FGR and PointNetLK, were more sensitive, with the recall metric decreasing from 50% to 10% and 90% to 0% as the bounding box was increased by 80%. Meanwhile, RPMNet and RANSAC converged more slowly to a lower recall value. GO-ICP, on the other hand, no longer converged when the bounding box was increased. As a general guideline, the best results were obtained when the object’s information was maximized and the environmental clutter was minimized. Thus, pre-processing is important to a successful alignment. Furthermore, this limits the maximal size of the bounding box extracted from preliminary object detection steps.

For the training validation, the lower recall values can be explained by the scaling. The limit for recall was chosen at 1 cm, while the average translational error was larger in these cases. Additionally, the $R^{2}$ metric is more sensitive to numerical rounding errors, especially when working with larger values. Still, all other metrics showed the good performance of these methods on the synthetic datasets. On the point cloud scans, the normal datasets led to the best results in terms of accuracy. The Cranfield benchmark could generalize well, even to the considered ModelNet40 objects. Thus, adding Gaussian noise or partiality did not directly lead to improved results. This indicates the need for a better model of the captured point cloud scans to improve the training dataset.

For all methods, ICP refinement led to significant improvements over all objects, with rotational and translational errors decreasing more than $10^{\circ }$ and 5 mm, respectively. The only exception to this trend was GO-ICP, where the relative gain of applying refinement diminished from 90% and 80% to almost 0% for the MRAE and MRTE, respectively, when decreasing the MSE threshold. The highest recorded registration time for ICP was 0.32 s, for the largest object, but it typically ranged on the order of $10^{-2}$ s for most other cases.

### 4.2. PCR Methods Comparison

Of all methods, GO-ICP achieved the best performance in terms of precision. The major downside of GO-ICP lay in the higher registration time of multiple seconds up to a minute, making it insufficient for real-time applications. This agreed with the literature [8], where errors of around $5^{\circ }$ were obtained on the Stanford dataset. RMSEs were reported of at most 0.05 when applied on synthetic data, while we found errors of $0.04^{\circ }$ and 1.58 mm. ICP refinement only took 0.01 s on average, as there was only a small gain.

RANSAC, refined with ICP, reached similar performance to GO-ICP, while showing higher variances. This can be explained by the fact that RANSAC uses a random initialization which can cause different results for the same scan. However, RANSAC was much faster than GO-ICP, with average registration times below 1 s. Furthermore, parameters needed to be correctly set to achieve a high performance as indicated in the literature [41].

FGR was one of the quickest methods and had a similar performance to RANSAC’s, although it was less accurate and with more failure cases. The method is also more prone to noise, as indicated by [42]. RMSEs of only 0.008 were achieved on synthetic data, even when adding noise with σ=0.005. On a scene benchmark, a recall of only 51.1% was obtained, which was lower than 61.18% found in this paper. We found speeds for FGR around 0.1 s, which was close to the reported 0.2 s in the literature [42].

PointNetLK achieved the best performances overall for the learning-based methods. Like RANSAC and FGR, the results were the best when PointNetLK was refined using ICP. However, the number of failure cases and variability between the results were both higher. PointNetLK yielded the same result for a single scan, leading to slightly lower deviations than RANSAC and FGR. Registration times were limited to 0.12 s and GPU requirements were the lowest among the tested learning-based methods. Still, PointNetLK required an extensive training process with limited ability to generalize to unseen data. Furthermore, the performance dropped when transformations were larger, as also indicated in the literature [43].

For RPMNet, the number of failure cases was lower than for PointNetLK and FGR, showing the higher robustness to initialization, as mentioned in the literature [9]. RPMNet converged more easily on complex datasets compared to PointNetLK. However, GPU requirements were also higher. RPMNet achieved errors smaller than a unit degree or millimetre when applied to clean data. Errors increased slightly when Gaussian noise was added, but were still much below $25.78^{\circ }$ and 10.21 mm found on 3D scans.

ROPNet was outperformed by the other methods, with an MRAE of $7.90^{\circ }$ and MRTE of 4.89 mm and around 70% failure cases, after refinement. The performance was lower than reported in the literature [44]. On synthetic data, ROPNet could achieve errors of around $1^{\circ }$, even on unseen ModelNet40 data with added noise. This can be related to the datasets not representing the real-world scans sufficiently well. As a result, ROPNet had difficulty generalizing to the scanned data.

Our results show that learning-based methods (PointNetLK, RPMNet) can match the performance of classical methods like RANSAC and FGR. However, the studied techniques also highlight the need to consider trade-offs in accuracy, speed and pre-processing. Practical considerations are further discussed in Section 4.3.

As a final observation, failures typically occurred due to a low point cloud quality, a too large initial transformation or due to the choice of parameters. As a result, we observed high variances over different objects. ICP could refine the successful registration results, but could not find large transformations that would turn a failure into a success. The lower the number of failure cases, the better the method can generalize to different objects and scans.

### 4.3. PCR Methods Guidelines

**Figure 11:**
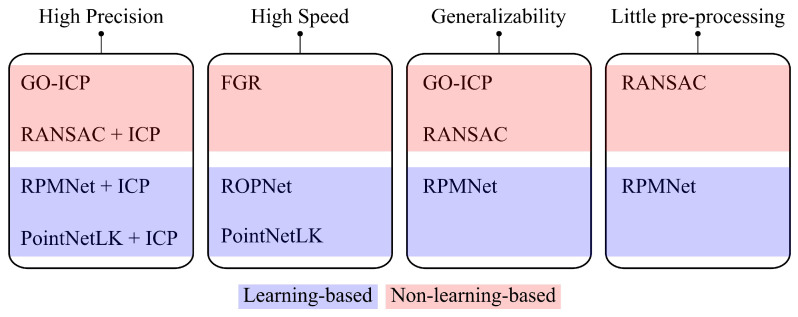
Overview of the compared methods, grouped according to their main strengths.

An overview of the compared methods is shown in Figure 11, which can serve as a simple guide to aid in selecting the correct registration method for the reader’s application.

RANSAC, PointNetLK and RPMNet combined with ICP refinement yield accurate results and fast registration times. However, errors are larger when applying the methods on real-world scans compared to applying them on synthetic data. These methods can be used in applications requiring a real-time estimation of the transformation matrix, while still achieving precise results on most scans. RANSAC and FGR, in particular, require no training and have low computation times and requirements. These methods are most interesting in real-time registration and could benefit from the knowledge of previously found transformations as an initial guess. RANSAC generalizes well to different objects and scans while being less sensitive to environmental clutter.

ROPNet and PointNetLK achieve high speeds after training. Furthermore, RPMNet can generalize to different objects and yields the best results among the tested methods when clutter is included in the point cloud scan. Learning-based methods need training, which requires more time and higher GPU requirements. Moreover, convergence during training is not guaranteed. Further investigation into their training process and ability to generalize are required to achieve high-fidelity results.

GO-ICP achieved the highest accuracies among the evaluated methods, even reaching similar performance to that found in the literature. However, the method was also the slowest, with registration times of multiple seconds. Furthermore, it required a cleaned-up point cloud with limited outliers and environmental clutter. GO-ICP should be considered for applications requiring high-accuracy results, where computation times do not play a significant role, like 3D modelling.

### 4.4. Limitations

The present study included only a limited number of registration methods. Future work could focus on extending the comparison, to include state-of-the-art methods such as DeepPro [39], REGTR [40] or TEASER++ [6]. Additionally, classical probabilistic methods, like NDT or CPD, can also be evaluated.

The ground-truth estimation only allowed us to estimate the registration accuracy up to a couple of millimetres and degrees, which might be insufficient for more sensitive applications. Furthermore, differences between methods of less than 2 mm or $3^{\circ }$ cannot be considered significant.

The quality of the captured point cloud can be improved by using multiple cameras or a moving one, reducing the partiality in the data. Additionally, filtering [55,56,57,58] or point cloud completion [59] techniques can also be added.

Finally, the learning-based methods were trained on a series of basic datasets. Further investigation into training hyperparameters and more complex training datasets is required to achieve higher performance from the learning-based methods. Thus, these methods might achieve higher accuracies, after a more involved training process.

## 5. Conclusions

Practical applications of point cloud registration still largely rely on classical methods, like ICP. To accelerate the deployment of advanced methods, quantitative validations are essential. This study performed an in-depth comparison of six registration methods, focusing on classical techniques and deep-learning-based solutions.

Furthermore, literature reviews on point cloud registration algorithms are typically performed on synthetic datasets, instead of 3D scans. This paper compared six registration methods, including GO-ICP, RANSAC, FGR, PoinNetLK, RPMNet and ROPNet. Registration was performed on two point clouds, one sampled from a 3D CAD model and the other captured using the Intel RealSense D435i camera.

GO-ICP, as well as RANSAC, RPMNet and PointNetLK combined with ICP, achieved high-precision alignments, with small rotational and translational errors. Refinement had little effect on GO-ICP, hence it was not required. FGR, ROPNet and PointNetLK ranked among the fastest methods tested, each leading to registration times far below 1 s. GO-ICP, RANSAC and RPMNet led to the fewest failure cases, indicating their ability to work robustly for a wide array of scans and objects. Finally, RANSAC and RPMNet showed the most robustness to environmental clutter, thus requiring less pre-processing for the input point clouds.

Our results can be used by novice researchers in the field to select a PCR method for their application, based on quantitative metrics. Furthermore, the dataset and code used during the experiments have been made available to encourage new validation studies and comparisons.

Future work could focus on improving the capture of the point clouds. Here, a single camera was used at a fixed perspective. Instead, multiple cameras or a moving one can capture a more complete point cloud. Advanced filtering techniques can be considered to improve the point cloud quality. Finally, other methods such as DeepPro and Teaser++ show promising results and can be further tested using real-world data.

## Figures and Tables

**Table 1:** Parameters checked for each method. A check mark (✓) or range indicates the parameter is verified, a dash (/) means the parameter is not applicable and a lightning symbol (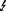) indicates the parameter is checked, but no convergence was reached. Training models are reported in Section 2.3.

Method	GO-ICP	RANSAC	FGR	PointNetLK	RPMNet	ROPNet
Zero mean	✓	✓	✓	✓	✓	✓
Refinement	✓	✓	✓	✓	✓	✓
Bounding box	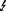	1→1.8				/
Voxel size [m]	$10^{-3},10^{-2}$	$10^{-4}\to 10^{-1}$		$0,10^{-3}\to 10^{-2}$		
MSE Threshold [m]	$10^{-5}\to 10^{-1}$	/	/	/	/	/
Trim fraction [/]	$10^{-4}\to 10^{-1}$	/	/	/	/	/
Training model	/	/	/	✓	✓	✓

**Table 2:** Validation results of training PointNetLK, RPMNet and ROPNet on (1): normal data, (2): noisy data, (3): partial data, (4): floor, noisy and partial data. Training–test iterations were 820 and 205, respectively. The data used for training are also mentioned (A: all, L: limited). The recall limit was 0.01 m. The arrows indicate whether the metric should be as small (↓) or high (↑) as possible for a good registration result.

Dataset	Data	MRAE	MRTE	RMSE	RMSE	MAE	MAE	Recall	$R^{2}$
	[A/L]	[°] ↓	[mm] ↓	[°] ↓	[mm] ↓	[°] ↓	[mm] ↓	[%] ↑	[/] ↑
PointNetLK									
1	A	2.13	0.78	0.07	0.05	0.37	0.00	91.71	1.00
2	L	0.31	2.94	0.00	2.88	0.13	0.01	97.82	1.00
RPMNet									
1	A	2.60	3.00	0.04	2.23	0.82	0.00	70.76	1.00
2	A	2.90	3.16	0.05	2.14	0.67	0.00	72.82	1.00
3	A	2.71	16.42	0.04	10.52	0.85	0.11	49.78	1.00
4	A	2.40	25.76	0.02	17.94	0.98	0.32	30.40	1.00
ROPNet									
1	A	0.02	0.00	0.00	0.00	0.00	0.00	100.00	1.00
2	A	0.24	1.76	0.00	1.22	0.11	0.00	99.91	1.00
3	A	0.99	8.83	0.02	11.95	0.49	0.14	79.67	1.00
4	A	1.12	11.51	0.01	7.33	0.48	0.05	69.58	1.00

**Table 3:** For each criterion, a representative metric was chosen. ICP refinement was not applied during the experiments for pre-processing and speed. However, ICP refinement was included for all other values. Dash (/) indicates missing data. Lightning (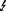) indicates no convergence.

Method ↓	Precision	Variance	Speed	Generalizability	Pre-Processing
Metric →	R^2^ ↑	*σ*(R^2^) ↓	Time [s] ↓	Failure [%] ↓	Recall [%] for *BB* = 1.8
GO-ICP	0.98	0.04	15.50	28.64	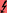
RANSAC	0.93	0.16	0.27	43.58	21.70
FGR	0.81	0.20	0.10	47.26	12.65
PointNetLK	0.97	0.15	0.12	58.65	0
RPMNet	0.89	0.22	0.12	45.52	48.87
ROPNet	0.95	0.12	0.06	70.60	/

## Data Availability

The Python code, used to run the experiments, as well as the point clouds scans with their ground truth, is available at https://github.com/Menthy-Denayer/PCR_CAD_Model_Alignment_Comparison.git (accessed on 14 February 2024).

## Appendix A. Ground-Truth Validation

We verified the ground truth by applying ICP refinement to the found transformation, similarly to [20]. Table A1 gives an overview of the results, where a voxel size of 1 cm was used to run the ICP algorithm. The metrics showed a small correction made by ICP, indicating a good ground-truth definition. Hence, we used the results without refinement for the experiments.

**Table A1:** Mean errors (ϵ) and standard deviations (σ), when applying ICP to refine the found ground-truth estimation, for different bounding boxes (BB). A voxel size of 1 cm was used for ICP.

Type	Data	MRAE	MRTE	RMSE	RMSE	MAE	MAE	Recall	$R^{2}$
	[*ϵ*/*σ*]	[°] ↓	[mm] ↓	[°] ↓	[mm] ↓	[°] ↓	[mm] ↓	[%] ↑	[/] ↑
Cleaned point clouds	ϵ	1.85	1.89	0.02	1.09	2.69	0.00	100	1.00
	σ	1.22	1.11	0.01	0.64	7.93	0.00	0.00	0.01
BB=1.0	ϵ	1.50	2.53	0.01	1.46	0.70	0.00	100	1.00
	σ	0.88	1.66	0.01	0.96	0.83	0.01	0.00	0.00
BB=1.2	ϵ	1.24	2.93	0.01	1.69	0.64	0.00	100	1.00
	σ	0.66	1.48	0.01	0.85	0.86	0.00	0.00	0.01
BB=1.4	ϵ	1.50	2.97	0.01	1.71	0.71	0.00	100	1.00
	σ	0.87	1.62	0.01	0.94	1.07	0.00	0.00	0.00
BB=1.6	ϵ	1.58	3.57	0.01	2.06	0.73	0.01	100	1.00
	σ	0.96	2.34	0.01	1.35	1.07	0.01	0.00	0.00
BB=1.8	ϵ	1.58	3.36	0.01	1.94	0.75	0.01	100	1.00
	σ	0.93	2.17	0.01	1.25	1.08	0.01	0.00	0.00

## Appendix B. List of Averaged Metrics

**Table A2:** Metrics (ϵ) and standard deviations (σ) for the studied registration methods. Recall was taken with a 0.01 m threshold. The best metrics are shown in **bold**. The time mentioned for the refinement cases only includes ICP.

Method	Data	MRAE	MRTE	RMSE	RMSE	MAE	MAE	Recall	$R^{2}$	Failure	Time
	[*ϵ*/*σ*]	[°] ↓	[mm] ↓	[°] ↓	[mm]↓	[°] ↓	[mm]↓	[%]↑	[/]↑	[%] ↓	[s] ↓
Non-learning-based methods											
GO-ICP	ϵ	**4.85**	2.73	**0.04**	1.58	4.00	**0.00**	**100**	**0.98**	**28.64**	15.50
	σ	3.08	1.61	0.02	0.93	4.13	0.01	0.00	0.04	30.08	23.14
GO-ICP + ICP	ϵ	4.85	**2.73**	0.04	**1.58**	**3.99**	0.00	100	0.98	28.64	**0.01**
	σ	3.10	1.58	0.03	0.91	4.14	0.01	0.00	0.04	30.08	0.00
RANSAC	ϵ	18.62	6.23	0.16	3.60	16.43	0.02	85.94	0.88	50.82	0.27
	σ	12.64	4.20	0.09	2.42	31.31	0.03	21.71	0.13	26.24	0.22
RANSAC + ICP	ϵ	5.85	2.83	0.05	1.63	9.70	0.01	97.34	0.93	43.58	0.04
	σ	7.20	2.51	0.06	1.45	29.05	0.02	9.52	0.16	24.63	0.02
FGR	ϵ	27.41	11.67	0.29	6.74	19.08	0.06	61.18	0.58	75.57	**0.10**
	σ	38.52	4.56	0.13	2.63	14.14	0.06	27.50	0.20	17.52	0.17
FGR + ICP	ϵ	12.59	4.95	0.10	2.86	7.81	0.01	91.26	0.81	47.26	0.04
	σ	12.26	3.40	0.10	1.96	7.00	0.02	13.62	0.20	11.46	0.03
Learning-based registration											
PointNetLK	ϵ	14.04	6.36	0.11	3.67	12.55	0.02	95.18	0.86	57.92	0.12
	σ	13.86	1.09	0.11	0.63	34.82	0.01	12.44	0.16	34.05	0.06
PointNetLK + ICP	ϵ	**4.12**	**2.36**	**0.03**	**1.36**	7.80	**0.00**	**99.57**	**0.97**	58.65	0.05
	σ	8.55	1.31	0.07	0.76	36.18	0.00	2.95	0.15	28.24	0.03
RPMNet	ϵ	25.78	10.21	0.21	5.90	11.08	0.04	71.48	0.62	59.31	0.12
	σ	7.77	1.09	0.06	0.63	2.75	0.01	14.83	0.14	25.20	0.09
RPMNet + ICP	ϵ	7.59	3.65	0.06	2.11	**4.52**	0.01	95.94	0.89	**45.52**	**0.04**
	σ	8.05	2.80	0.06	1.62	6.61	0.01	9.31	0.22	30.34	0.03
ROPNet	ϵ	25.88	10.99	0.21	6.35	30.84	0.05	67.38	0.72	74.70	**0.06**
	σ	10.22	2.19	0.08	1.27	9.32	0.02	6.50	0.08	16.90	0.02
ROPNet + ICP	ϵ	7.90	4.89	0.06	2.82	31.91	0.01	94.33	0.95	70.60	**0.04**
	σ	12.31	3.30	0.09	1.91	8.76	0.02	6.29	0.12	17.01	0.05
